# TensorFlow-based MobileNetV2 U-Net tumor segmentation and multiparametric MRI radiomics for predicting cervical lymph node metastasis in oral tongue squamous cell carcinoma

**DOI:** 10.1177/17588359261421325

**Published:** 2026-02-10

**Authors:** Qiangqiang Gang, Jie Feng, Bingmei Chen, Na Zhang, Ke Zhang

**Affiliations:** Department of Radiology, Southern Medical University Nanfang Hospital, Guangzhou, China; Department of Radiology, Southern Medical University Nanfang Hospital, Guangzhou, China; Department of Radiology, Southern Medical University Zhujiang Hospital, Guangzhou, China; Paul C. Lauterbur Research Center for Biomedical Imaging, Shenzhen Institute of Advanced Technology, Chinese Academy of Sciences, Shenzhen, China; Department of Diagnostic and Interventional Radiology, Heidelberg University Hospital, Heidelberg 69120, Germany

**Keywords:** lymph node metastasis, MobileNetV2 U-Net, MRI, oral tongue squamous cell carcinoma, radiomics

## Abstract

**Background:**

In oral tongue squamous cell carcinoma (OTSCC) patients, cervical lymph node metastasis profoundly influences prognoses and is central to guiding surgical strategies. Mapping the likelihood of lymph node metastasis across different cervical nodal levels is essential for achieving precise surgical planning.

**Purpose:**

OTSCC is a prevalent head and neck malignancy. Accurate MRI-based tumor segmentation and prognostic prediction are essential for detecting lymph node metastasis and improving patient survival rates. However, the potential of deep learning techniques has been underexplored in this context.

**Design:**

This retrospective pilot study included 136 OTSCC patients with non-lymph node metastasis and lymph node metastasis who underwent primary and cervical lymph node dissection following baseline MRI. The development of a machine learning approach, incorporating an automatically segmented approach, enables the creation of a model capable of predicting cervical lymph node metastasis based on primary site tumors.

**Methods:**

We propose a two-stage OTSCC diagnostic workflow. First, a multiparametric fusion network (MobileNetV2 U-Net) was implemented using TensorFlow to integrate features from contrast-enhanced T1-weighted (CE-T1WI), T2-weighted (T2WI), and T1-weighted (T1WI) MRI sequences and automatically segment primary tumors. Next, radiomic models were constructed to predict cervical lymph node metastasis from these automated segmentations. A fusion nomogram combining radiomic features and clinical data was developed to predict metastasis status. For comparison, a radiomics model using manually segmented CE-T1WIs was also evaluated.

**Results:**

Data from 136 patients (mean age 50.29 ± 12.25 years; 100 men, 36 women) showed that the MobileNetV2 U-Net achieved a Dice similarity coefficient (DSC) of 85% and a mean intersection over union (IoU) of 76% on the training set, and a DSC 87% and an IoU 79% on the independent test set. The fusion nomogram achieved areas under the ROC curves of 0.98 and 0.93 on the training and test sets, respectively, when using the automated segmentation masks. The automated segmentation nomogram performed comparably to the model using manual segmentations for predicting lymph node metastasis.

**Conclusion:**

Our TensorFlow-based MobileNetV2 U-Net provides clinicians with an automated tool to delineate OTSCC tumors and predict cervical lymph node metastasis, potentially aiding personalized surgical planning.

## Introduction

In Oral tongue squamous cell carcinoma (OTSCC) patients, cervical lymph node metastasis profoundly influences prognoses and is central to guiding surgical strategies.^1^ Evidence suggests that elective neck dissection in clinically negative (cN0) early-stage OTSCC patients significantly mitigates the risk of occult metastasis, thus improving survival outcomes. However, because approximately 30%–50% of cN0 patients lack actual nodal involvement, elective procedures often result in avoidable trauma and morbidity.^2–4^

Mapping the likelihood of lymph node metastasis across different cervical nodal levels is essential for achieving precise surgical planning. Studies have consistently shown that OTSCC metastasis predominantly involves levels II and III nodes, thus informing standard neck dissection protocols, especially in more advanced T3 and T4 patients.^2,3^ In addition to anatomic markers,^5,6^ molecular profiles, such as high expression levels of CDKN2A and PLAU, are emerging as robust predictors of lymph node metastasis in OTSCC patients.^7^ Predictive models that integrate these biomarkers with pathological grades and tumor stages offer promising avenues for implementing individualized risk stratification and refined surgical planning.^8,9^

The concept of individualized surgical approaches is gaining attraction in OTSCC management scenarios, as it aligns treatments with tumor biology and patient-specific disease progression trends.^10,11^ Integrating molecular diagnostics and advanced imaging techniques could optimize the surgical scope, increasing both the survival rate and the quality of life for OTSCC patients.^3,8^

The acquisition of extensive data from MR images, which enables signal extraction and enhancement pattern analyses, has garnered increasing interest within the scientific community. This approach allows for the derivation of biological data from MR images through mathematical analyses, providing a noninvasive, cost-effective, and time-efficient alternative that poses no risk to patients.^12–15^ In this study, multiparametric radiomics data were used to assess segmentations derived from preoperative MR images generated by a MobileNetV2 U-Net model to develop nomograms for predicting cervical lymph node metastasis statuses in tongue cancer patients on the basis of their primary tumor lesions.

## Materials and methods

### Datasets and implementations

The institutional review board approved this retrospective study (NFEC-2022-328), and the requirement for informed consent was waived. The reporting of this study conforms to the STARD statement. A total of 136 patients with OTSCC who were retrospectively admitted to Nanfang Hospital, Southern Medical University, between October 10, 2016 and June 26, 2023 and Zhujiang Hospital, Southern Medical University, between June 10, 2019 and December 14, 2024 and who underwent MRI examinations before surgery were included. The inclusion criteria were as follows: (1) patients whose OTSCCs were verified by pathology; (2) patients at the clinical tumor stage (T stage; T1–T4); (3) patients who had no previous/synchronous tumors; (4) patients who had no previous neck surgery; (5) patients who had dissection for at least three contiguous neck node levels; (7) patients who had lymph node surgery performed at Nanfang Hospital; and (8) patients who had neck specimens processed at surgical levels in the standard manner. MRI was performed using various high-field scanners, including PHILIPS Ingenia 3.0T (Philips Healthcare, Best, The Netherlands), GE HDxt 3.0T (GE Healthcare, Waukesha, WI, USA), PHILIPS Achieva Tx 3.0T (Philips Healthcare, Best, The Netherlands), GE Signa HDx Series 1.5T (GE Healthcare, Waukesha, WI, USA), and SIEMENS Prisma 3.0T (Siemens Healthcare GmbH, Erlangen, Germany). The imaging protocols were tailored to each system to optimize the diagnostic quality level. The specific MRI scanning parameters for T1-weighted (T1WIs), T2-weighted (T2WIs), and contrast-enhanced T1-weighted (CE-T1WIs) are provided in the Supplemental Materials.

The MR images were annotated by two radiologists via X-AnyLabeling (https://github.com/CVHub520/X-AnyLabeling) and 3D-Slicer (https://www.slicer.org). One radiologist with 10 years of experience was responsible for providing semiautomatic annotations, whereas another radiologist with 20 years of experience reviewed these annotations. Discrepancies were discussed further until an agreement was reached.

### Overall design

In this paper, we propose a two-stage workflow for diagnosing OTSCC. First, a multiparameter fusion MobileNetV2 U-Net integrated features from CE-T1WIs, T2WIs, and T1WIs to automatically segment tumors. In the second stage, a multiparametric radiomic model leveraged these segmentations to predict lymph node metastasis. Finally, a fusion nomogram incorporating radiomics features and clinical data was developed to enhance the metastasis prediction process.

### Automatic segmentation of multiparametric MR images

In this phase, we propose a comprehensive solution for conducting lesion segmentation in OTSCC patients. Our segmentation phase was based on multiparametric magnetic resonance images (CE-T1WIs 607 images, T2WIs 554 images, and T1WIs 559 images) and annotations. The model input consisted of slices derived from the same plane of the CE-T1WI sequence, along with slices from the T2WI and T1WI sequences. A simple data augmentation process was performed by randomly flipping the images (Figure S1).

The model used in this study was a modified MobileNetV2 U-Net.^16^ A pretrained MobileNetV2 model was used as the encoder.^17^ For the decoder, upsampling blocks were employed, which were implemented in pix2pix based on a conditional generative adversarial network (cGAN); this network learned the mapping from the input images to the output images.^18,19^ The structure of the segmentation architecture consists of the following modules: an encoder, MobileNetV2 U-Net, a cGAN, and a decoder. The complete code is available at https://www.tensorflow.org/tutorials/images/segmentation?hl=zh-cn. A batch size of two was used, and the model was trained for 40 epochs while monitoring its performance on the validation set. Trimaps were obtained by the expansion corrosion method, and the separated masks were stacked to obtain voxels of interests (VOIs). To evaluate the performance of the model, their training and validation losses, dice similarity coefficient (DSC), mean intersection over unions (IoUs), and accuracy values were monitored. All the experiments were conducted on a system equipped with an NVIDIA Tesla T4 GPU with 14.63 GB of available memory, and TensorFlow version 2.17.1 and Python 3.10.12 were utilized. The GPU was enabled for deep learning tasks, ensuring efficient computations and model training processes. Code can be found on the web page https://github.com/roseblingbling/U-net-segmentation/tree/main.

### Radiomics model establishment phase using manual segmentation on CE-T1WI MR images and automated segmentation of the multiparametric MR images from the previous stage

In this stage, the segmentation masks obtained from the previous stage for CE-T1WI, T2WI, and T1WI sequences and the manual segmentation masks obtained from CE-T1WI sequences, along with their clinical characteristics, were analyzed in a multifactorial manner to predict patients’ lymph node metastasis statuses. The workflow for predicting prognoses is shown in the Supplemental Material (Figure S7) and is described in detail below.

We selected 88 cases as the training dataset (36/52 = positive/negative). We also selected another 48 cases as the independent testing dataset (22/26 = positive/negative). After conducting *Z*-score normalization, five radiomics models were established; the feature selection methods and classifiers for the five radiomics models can be seen in Supplemental Material (Table S1). Logistic regression models were used to construct nomograms.

The areas under the ROC curves (AUCs) were calculated for quantification purposes and were compared using the DeLong test. The accuracy, sensitivity, specificity, recall, F1 score, and precision metrics were also calculated at a cutoff value that maximized the value of the Youden index. We also estimated the 95% confidence interval (CI) by performing bootstrapping with 1000 samples. All the above processes were implemented with FeAture Explorer Pro (FAEPro, V 0.3.6)^20^ in Python (3.7.6).

## Results

The final cohort size obtained after the application of the inclusion/exclusion criteria was 136 (mean age: 50.29 ± 12.25, 100 men, 36 women).

Table 1 presents the clinical features that were related to OTSCC lymph node metastasis based on the medical records of the included patients. Table 1 shows no significant gender, age, betel nut history, TNM stage, or depth of invasion differences between the training and testing groups.

**Table 1.** Clinical characteristics of 136 patients with OTSCC.

Patient description	Train set, *n* (%)	Test set, *n* (%)	*p* Value
Gender			0.91
Men	65 (73.9)	35 (72.9)	
Women	23 (26.1)	13 (27.1)	
Age	49.90 ± 12.66	51.02 ± 11.57	0.60
Areca-nut	20 (22.7)	6 (12.5)	0.15
N stage	40 (45.5)	22 (45.8)	0.46
N0	48 (54.5)	26 (54.2)	
N1	19 (21.6)	3 (6.25)	
N2	20 (22.7)	19 (39.6)	
N3	1 (1.1)	0 (0)	
M stage	0 (0)	0 (0)	N/A
T stage			0.93
T0	0 (0)	2 (4.2)	
T1	5 (5.68)	4 (8.33)	
T2	40 (45.5)	20 (41.7)	
T3	30 (34.1)	8 (16.7)	
T4	13 (14.8)	14 (29.2)	
DOI (cm)	1.32 ± 0.74	1.41 ± 0.71	0.47

DOI, depth of invasion; OTSCC, oral tongue squamous cell carcinoma; N/A, not available.

### Automatic segmentation of the OTSCC tumor region

**Figure 1.**
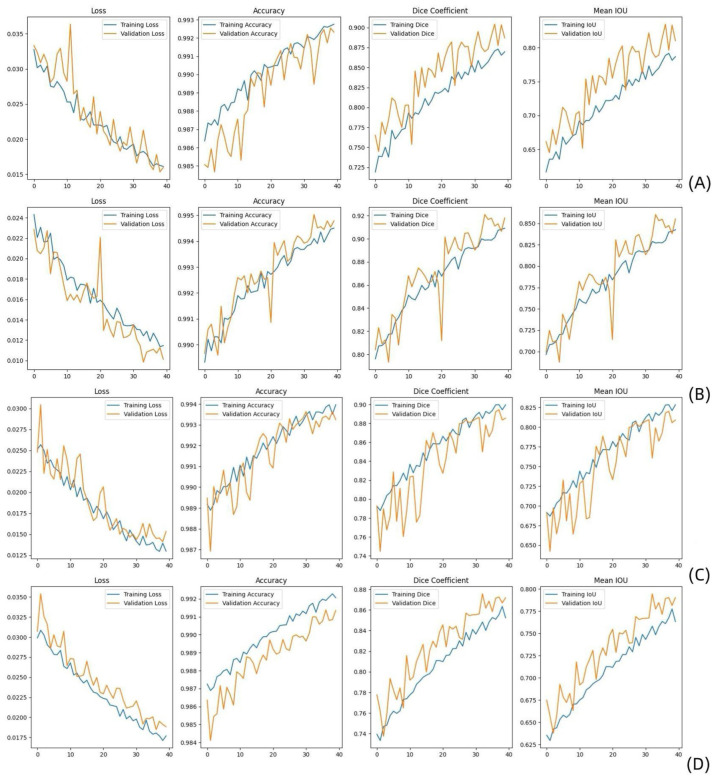
Performance comparison between modules using multi and single-parameter MR images as their inputs during the training and testing phases implemented on T1WIs (a), T2WIs (b), CE-T1WIs (c), and T1WI + T2WI + CE-T1WIs (d). Metrics such as the loss, accuracy, Dice coefficient, and mean IoU reflect the ability of each model to effectively segment tumor regions. CE-T1WI, contrast-enhanced T1-weighted; IoU, intersection over union; T2WI, T2-weighted.

**Figure 2.**
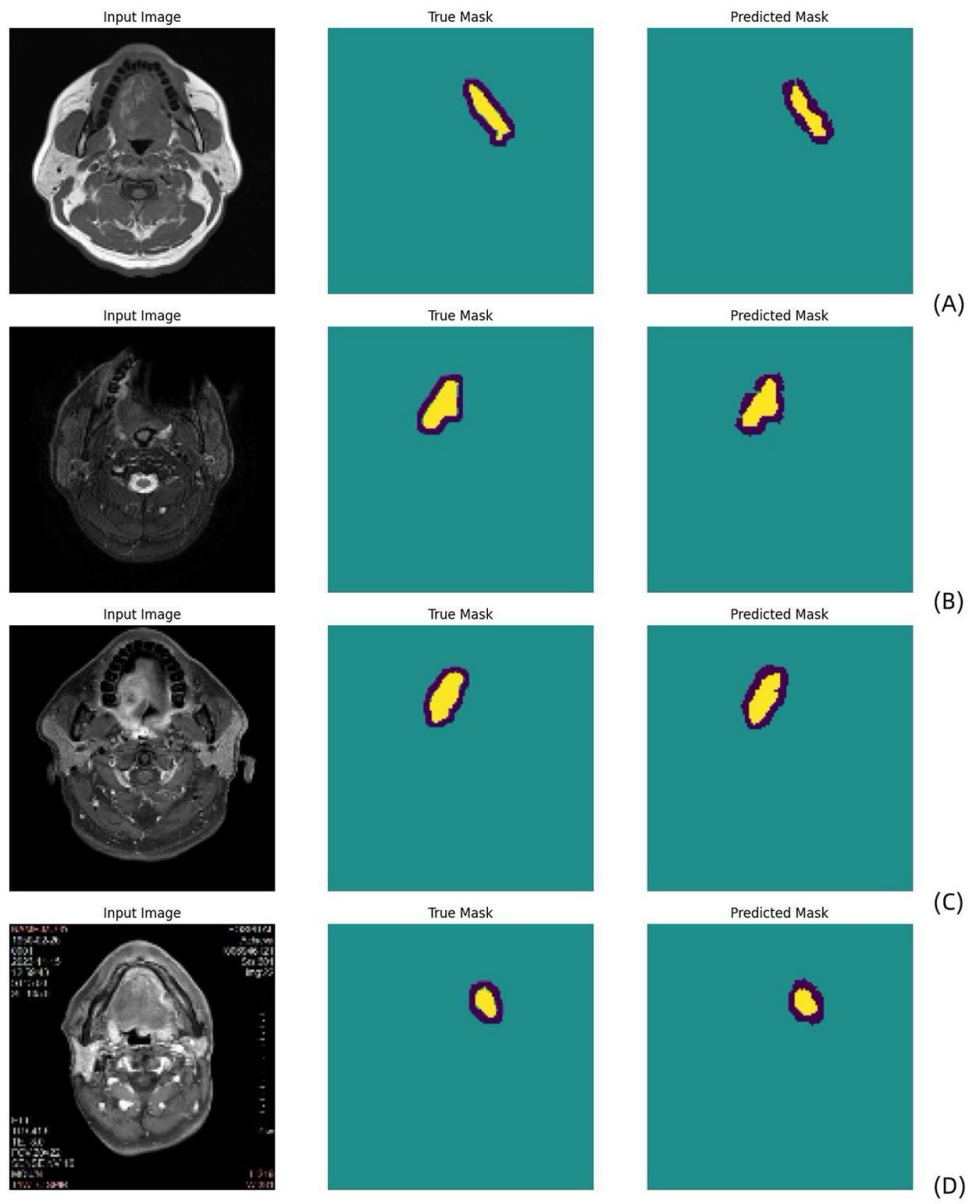
Schematic diagram of the MobileNetV2 U-Net segmentation process conducted on T1WI (a), T2WI (b), CE-T1WI (c), and multiparametric MR sequences (d) (blue: uncertain regions; yellow: tumor regions). The true mask is the ground truth manually defined by the radiologist, and the predicted mask is the automatic MobileNetV2 U-Net segmentation result. CE-T1WI, contrast-enhanced T1-weighted; T2WI, T2-weighted.

A detailed analysis of the results obtained on the training and validation sets was conducted. Table 2 shows the performance comparison between the results obtained when using multi- and single-parameter MR sequences as model inputs during the training and testing phases. According to the experimental results, the segmentation performance was better when multiparametric MR images were used as the inputs for the training and test sets (mean IoU: 76%/85% DSC and mean IoU: 79%/87% DSC, respectively). For T1WIs, the results were (mean IoU: 78%/87% DSC and mean IoU: 81%/89% DSC) on the training and testing sets, respectively. T2WIs and CE-T1WIs yielded the best performance. For T2WIs, the results were (mean IoU: 84%/91% DSC and mean IoU: 86%/92% DSC) on the training and test sets, respectively; for CE-T1WIs, the results were (mean IoU: 83%/90% DSC and mean IoU: 81%/89% DSC) on the training and test sets, respectively. Due to the poor lesion recognition of TWI, the segmentation accuracy of multiparameter MRI was lower than that of T2WI and CET1. In general, multiparametric MRI provides more comprehensive imaging information, which can improve the predictive model’s performance. Figure 1(a)–(d) shows that the model performed well during training. Both the DSC and the mean IoU indicators demonstrated good performance. Figure 2(a)–(d) shows a schematic of the MobileNetV2 U-Net segmentation process. As shown in Figure 2(a)–(d), our model was able to accurately segment the tumor region.

**Table 2.** Segmentation performance achieved for the OTSCC tumor regions by using manual segmentations of CE-T1WI images and segmentations obtained from the previous stage for multiparametric MR images as inputs during both the training and validation phases.

Variables	Accuracy	DSC	Loss	Mean IoU
T1WI				
Train	0.99	0.87	0.02	0.78
Test	0.99	0.89	0.016	0.81
T2WI				
Train	0.99	0.91	0.01	0.84
Test	0.99	0.92	0.01	0.86
CET1WI				
Train	0.99	0.90	0.01	0.83
Test	0.99	0.89	0.02	0.81
T1 + T2 + CET1				
Train	0.99	0.85	0.02	0.76
Test	0.99	0.87	0.02	0.79

CE-T1WI, contrast-enhanced T1-weighted; DSC, dice similarity coefficient; Mean IoU, mean intersection over union; OTSCC, oral tongue squamous cell carcinoma; T2WI, T2-weighted.

### Radiomics model based on automated segmentation for multiparametric MR images

**Figure 3.**
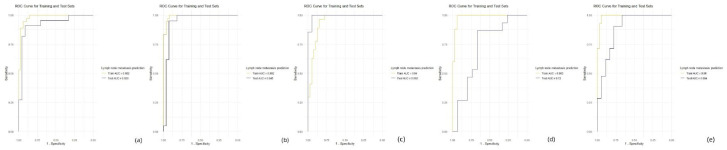
ROC curves produced by the OTSCC lymph node metastasis prediction models established using automatic segmentations obtained from the previous stage for multiparametric MR images (a) and manual segmentations of CE-T1WIs (b) in both the training and validation phases. Single-parameter radiomics model performances achieved based on automated segmentation, including T1WI, T2WI, and CET1 models (c)–(e), in both the training and validation phases. CE-T1WI, contrast-enhanced T1-weighted; OTSCC, oral tongue squamous cell carcinoma; T2WI, T2-weighted.

The signatures with the radiomics features and the clinical combination yielded AUCs of 0.98 (95% CI: 0.96–1) and 0.93 (95% CI: 0.84–1) on the training and test sets, respectively (Figure 3(a)). The feature contributions and their ranks are shown in the Supplemental Material (Figures S3–S6), and the prediction outcomes provided by this model across different sets are shown in Table 3. The performance of single-parameter radiomics models based on automatic segmentation, including those using T1WIs, T2WIs, and CET1 images, is shown in Table 4 and Figure 3(c)–(e).

**Table 3.** Performance of the clinical radiomics OTSCC lymph node metastasis prediction nomograms established using manual segmentations of CE-T1WIs and automatic segmentations obtained from the previous stage for multiparametric MR images in both the training and validation phases.

Segmentation	Accuracy	AUC (95% CI)	Precision	Recall	F1	Sensitivity	Specificity
Manual segmentation							
Train	0.94	0.99 (0.98–1)	0.96	0.94	0.95	0.94	0.94
Test	0.90	0.95 (0.87–1)	1	0.81	0.90	0.81	1
Auto-segmentation with multiparameter MRI sequences							
Train	0.94	0.98 (0.96–1)	0.96	0.94	0.95	0.94	0.94
Test	0.87	0.93 (0.84–1)	0.85	0.92	0.88	0.92	0.82

AUC, areas under the ROC curves; CE-T1WI, contrast-enhanced T1-weighted; OTSCC, oral tongue squamous cell carcinoma.

**Table 4.** Performance comparison between single-parameter MRI models (T1WI, T2WI, CET1, T1 + T2 + CET1) using segmentations obtained from the previous stage as inputs during both the training and validation phases.

Model	Accuracy	AUC (95% CI)	Precision	Recall	F1	Sensitivity	Specificity
T1WI							
Train	0.85	0.94 (0.89–0.99)	0.88	0.88	0.88	0.88	0.81
Test	0.94	0.99 (0.97–1)	1	0.89	0.94	0.89	1
T2WI							
Train	0.93	0.98 (0.96–1)	0.94	0.94	0.94	0.94	0.92
Test	0.60	0.72 (0.53–0.91)	0.59	0.67	0.63	0.67	0.53
CET1							
Train	0.94	0.99 (0.97–1)	0.94	0.94	0.94	0.94	0.93
Test	0.77	0.88 (0.77–0.99)	0.74	0.78	0.76	0.78	0.76
T1 + T2 + CET1							
Train	0.94	0.98 (0.96–1)	0.96	0.94	0.95	0.94	0.94
Test	0.87	0.93 (0.84–1)	0.85	0.92	0.88	0.92	0.82

AUC, areas under the ROC curves; CE-T1WI, contrast-enhanced T1-weighted; OTSCC, oral tongue squamous cell carcinoma; T2WI, T2-weighted.

**Figure 4.**
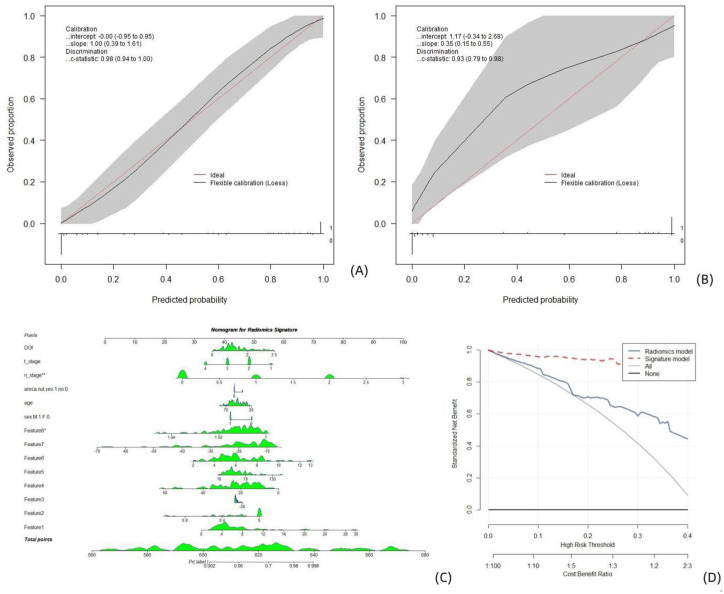
Clinical radiomics models established on segmentations obtained from the previous stage for multiparametric MR images. Calibration curves, nomograms, and decision analysis curves produced by the nomogram on the training set (a) and test set (b). (a, b) The *X*-axis is the nomogram-predicted probability of lymph node metastasis. The *Y*-axis represents the observed lymph node metastasis status, and the diagonal red line represents the ideal prediction for a perfect model. A nomogram (c) for predicting the risk of lymph node metastasis in patients with OTSCC. The value of each variable was given a score on the point scale axis. The total score was calculated by adding each individual score, and the total score was projected to the lower total score scale; that is, the probability of lymph node metastasis could be estimated. DCA curve (d), where the abscissa represents the threshold probability, and the ordinate represents the net benefit. All: the net benefit of assuming that all patients had lymph node metastasis. None: the net benefit of assuming that no patient had lymph node metastasis. Predicted probability: expected net benefit based on the predictive nomogram. DCA, decision curve analysis; OTSCC, oral tongue squamous cell carcinoma.

The calibration curve demonstrated optimal agreement between the predicted probabilities computed by the nomogram and the actual observations of the two patient cohorts (Figure 4(a) and (b)). The radiomic signatures and clinical records were included in the process of constructing the nomogram as predictors of lymph node metastasis in OTSCC patients (Figure 4(c)). A Decision Curve analysis (DCA; Figure 4(d)) revealed that the clinical “net benefit” was greater with this model than with the default strategy of treating all or none of the patients.

### Radiomics model based on manual segmentation for CE-T1W1 MR images

The signatures with the radiomics features and the clinical combination yielded AUCs of 0.99 (95% CI: 0.98–1) and 0.95 (95% CI: 0.87–1) on the training and test sets, respectively (Figure 3(b)). The feature contributions and coefficients are shown in the Supplemental Material (Figure S7), and the prediction outcomes provided by this model across different sets are shown in Table 3.

**Figure 5.**
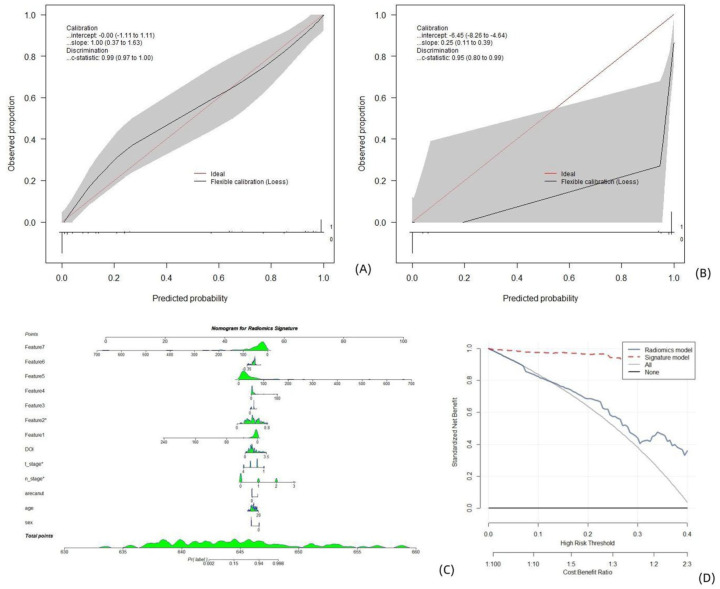
Clinical radiomics models established using manual segmentations obtained for CE-T1W1 MR images. Calibration curves, nomograms, and decision analysis curves produced by the nomogram on the training set (a) and test set (b). (a, b) The *X*-axis represents the nomogram-predicted probability of lymph node metastasis. The *Y*-axis represents the observed lymph node metastasis, and the diagonal red line represents the ideal prediction for a perfect model. A nomogram (c) for predicting the risk of lymph node metastasis in patients with OTSCC. The value of each variable was given a score on the point scale axis. The total score was calculated by adding each individual score, and the total score was projected to the lower total score scale; that is, the probability of lymph node metastasis could be estimated. DCA curve (d): The abscissa represents the threshold probability, and the ordinate represents the net benefit. All: the net benefit of assuming that all patients had lymph node metastasis. None: the net benefit of assuming that no patient had lymph node metastasis. Predicted probability: the expected net benefit based on the predictive nomogram. CE-T1WI, contrast-enhanced T1-weighted; OTSCC, oral tongue squamous cell carcinoma; T2WI, T2-weighted.

The radiomic signatures and clinical records were included in the process of constructing the nomogram as predictors of lymph node metastasis in OTSCC patients (Figure 5(c)). The calibration curve demonstrated optimal agreement between the predicted probabilities computed by the nomogram and the actual observations of the training patient cohort and relatively poor agreement with the test cohort (Figure 5(a) and (b)). A DCA (Figure 5(d)) revealed that the clinical “net benefit” was greater with this model than with the default strategy of treating all or none of the patients.

### Performance of the auto-segmented radiomics nomogram using multiple parameters: Comparison with manual segmented radiomics nomogram

(a) The AUC difference between the manually segmented radiomics nomogram and auto-segmented radiomics nomogram with multiple parameters in the test cohort was 0.249 (*p* = 0.804).
(b) The AUC difference between the manually segmented radiomics nomogram and auto-segmented radiomics nomogram with CET1 sequences in the test cohort was −0.481 (*p* = 0.632).
(c) The AUC difference between the auto-segmented radiomics nomogram with multiple parameters and auto-segmented radiomics nomogram with CET1 sequences in the test cohort was −0.399 (*p* = 0.690).

The AUC in the auto-segmented radiomics nomogram with multiple parameters was significantly higher than the AUC in the auto-segmented radiomics nomogram with T2WI sequences, with an AUC difference of 2.190 in the test cohort (*p* = 0.029). The AUC in the auto-segmented radiomics nomogram with multiple parameters was significantly higher than the AUC in the auto-segmented radiomics nomogram with T1WI sequences, with an AUC difference of −1.663 in the test cohort (*p* = 0.096).

## Discussion

Our research provides a comprehensive approach for conducting lesion segmentation and lymph node metastasis prediction in OTSCC patients via multiparametric MR images. Our results revealed that the tumor segmentation method implemented on T2WI model (DSC of 0.91 on the training set and 0.92 on the test set; mean IoUs of 0.84 on the training set and 0.86 on the test set), followed by the CE-T1WIs achieved the best performance (DSC of 0.90 on the training set and 0.89 on the test set; mean IoUs of 0.83 on the training set and 0.81 on the test set). And the overall multiparameter MR image segmentation performance was approximately equal to the T1WI segmentation performance, with DSC of 0.85 on the training set and 0.87 on the test set, and mean IoUs of 0.76 on the training set and 0.79 on the test set (Table 2 and Figure 1).

Two radiomic nomograms incorporating both MRI-based features and clinical data were developed to predict preoperative lymph node metastasis statuses, with the goal of aiding in therapeutic decision-making processes. As for auto-segmented radiomics nomogram, the MRI-based multiparameter radiomics model performed better (with an AUC of 0.98 (95% CI: 0.96–1) on the training set and an AUC of 0.93 (95% CI: 0.84–1) on the test set) than did the single-parameter radiomics models, including the T2WI (*p* < 0.1) and T1WI (*p* < 0.1) model, the CET1 model performed approximates to multiple parameter model (with an AUC of 0.99 (95% CI: 0.97–1) on the training set and an AUC of 0.88 (95% CI: 0.77–0.99) on the test set), as shown in Table 4 and Figure 3.

Interestingly, relative to the predictive performance of the nomogram using manual segmentation on CE-T1WI MR images (with AUCs of 0.99 (95% CI: 0.98–1) on the training set and 0.95 (95% CI: 0.87–1) on the test set), the nomogram using segmentations obtained from the previous stage for multiparametric MR images also performed well (with AUCs of 0.98 (95% CI: 0.96–1) on the training set and 0.93 (95% CI: 0.84–1) on the test set), with no statistical difference (*p* > 0.05). And the performance of the nomogram using segmentations obtained from the previous stage for CET1 MR images was similar to that of the manual segmentation nomogram (with AUC of 0.99 (95% CI: 0.97–1) on the training set and 0.88 (95% CI: 0.77–0.99) on the test set), with no statistical difference (*p* > 0.05), which can be seen in Table 3 and Figure 3, suggesting that the MobileNetV2 U-Net model could replace traditional manual segmentation methods in the future, streamlining the workflow and enhancing the efficiency achieved in clinical settings. The results of this experiment prove that automatic segmentation can replace manual segmentation in the future, thus saving considerable human labor via the radiomics model. The MobileNetV2 U-Net algorithm is the most promising algorithm.

Machine learning and artificial intelligence (AI) models, such as convolutional neural networks are being increasingly employed in medical imaging tasks to enhance noninvasive risk assessments, particularly in terms of predicting lymph node metastasis.^21–25^ Kudoh et al.^12^ investigated [^(18)^F]-fluoro-2-deoxyglucose PET scans and linked metabolic activity with metastatic potential, achieving an AUC of 0.91. Liu et al.^13^ leveraged multimodal MRI radiomics, which achieved improved predictive power (with AUCs of 0.73–0.89) through sequence combination. Wang et al.^14^ combined MRI radiomics and deep learning to reveal subtle patterns beyond the detection capabilities of humans. Zhong et al.^15^ used a radiomics method and attained an AUC of 0.94, emphasizing the enhanced detection of metastatic nodes in clinical contexts. Rezayi et al.^23^ investigated the use of AI to process X-ray-oriented images for diagnosing COVID-19. The MobileNetV2 U-Net architecture was adopted as the core of their segmentation model. MobileNetV2 U-Net, which was proposed by Ronneberger et al.^16^ exhibits exceptional performance in medical image segmentation tasks, particularly those involving small datasets, due to its symmetric encoder–decoder structure and skip connections.^26^ Jiao et al.^27^ noted that multitask learning not only enhances the performance of models but also reduces the risk of overfitting, especially in medical image analysis scenarios, where data are often limited.

However, despite the good performance achieved by our model in segmentation and classification tasks, some challenges remain. First, owing to the limited sample size of the OTSCC image dataset, the generalizability of the model needs further improvement. Long et al.^28^ proposed fully convolutional networks, which offer an important reference for addressing image segmentation problems. Similar techniques could help further improve our segmentation model, particularly in small-sample training scenarios. Additionally, the model may encounter performance degradations in cases with complex backgrounds or blurred tumor boundaries. Future work could consider incorporating technologies such as DeepLabV3+,^29,30^ which uses dilated convolutions to attain enhanced segmentation performance, especially for tumor region extraction tasks implemented in complex backgrounds.

## Conclusion

In conclusion, this study demonstrates the potential of an OTSCC segmentation and classification model based on radiomics and MobileNetV2 U-Net architectures and validates the effectiveness of joint task learning. This study has certain limitations that should be acknowledged. Firstly, the sample size was relatively limited, which may restrict the statistical power and generalizability of the findings. Secondly, the data were collected from only two centers, which may introduce institutional biases. To enhance the robustness and generalizability of our predictive model, further validation using external cohorts from multiple institutions is warranted. Multicenter studies with larger and more diverse patient populations will be essential to confirm the stability and applicability of the proposed model across different clinical settings. Future research will focus on expanding the dataset, optimizing the generalization ability of the model, and integrating advanced techniques such as dilated convolutions and multiscale feature extraction to further improve its diagnostic accuracy.

## Supplemental Material

sj-doc-1-tam-10.1177_17588359261421325 – Supplemental material for TensorFlow-based MobileNetV2 U-Net tumor segmentation and multiparametric MRI radiomics for predicting cervical lymph node metastasis in oral tongue squamous cell carcinoma Supplemental material, sj-doc-1-tam-10.1177_17588359261421325 for TensorFlow-based MobileNetV2 U-Net tumor segmentation and multiparametric MRI radiomics for predicting cervical lymph node metastasis in oral tongue squamous cell carcinoma by Qiangqiang Gang, Jie Feng, Bingmei Chen, Na Zhang and Ke Zhang in Therapeutic Advances in Medical Oncology

sj-docx-2-tam-10.1177_17588359261421325 – Supplemental material for TensorFlow-based MobileNetV2 U-Net tumor segmentation and multiparametric MRI radiomics for predicting cervical lymph node metastasis in oral tongue squamous cell carcinoma Supplemental material, sj-docx-2-tam-10.1177_17588359261421325 for TensorFlow-based MobileNetV2 U-Net tumor segmentation and multiparametric MRI radiomics for predicting cervical lymph node metastasis in oral tongue squamous cell carcinoma by Qiangqiang Gang, Jie Feng, Bingmei Chen, Na Zhang and Ke Zhang in Therapeutic Advances in Medical Oncology

## Declarations

**Ethics approval and consent to participate:** The institutional review board of Nanfang Hospital, Southern Medical University, approved this retrospective study, and the requirement for informed consent was waived.

**Consent for publication:** Not applicable.

**Availability of data and materials:** The data that support the findings of this study are available upon request from the corresponding author.
