# Supplementary material for: TensorFlow-based MobileNetV2 U-Net tumor segmentation and multiparametric MRI radiomics for predicting cervical lymph node metastasis in oral tongue squamous cell carcinoma
Source: Ther Adv Med Oncol. 2026 Feb 10;18:17588359261421325. doi: 10.1177/17588359261421325 (PMC12891416; doi:10.1177/17588359261421325)
Supplement: sj-docx-2-tam-10.1177_17588359261421325 – Supplemental material for TensorFlow-based MobileNetV2 U-Net tumor segmentation and multiparametric MRI radiomics for predicting cervical lymph node metastasis in oral tongue squamous cell carcinoma [file sj-docx-2-tam-10.1177_17588359261421325.docx]

Figure S1. (a) shows some samples from the training data, while (b) displays the augmented data. Data augmentation was performed by applying random transformations to generate additional training data.


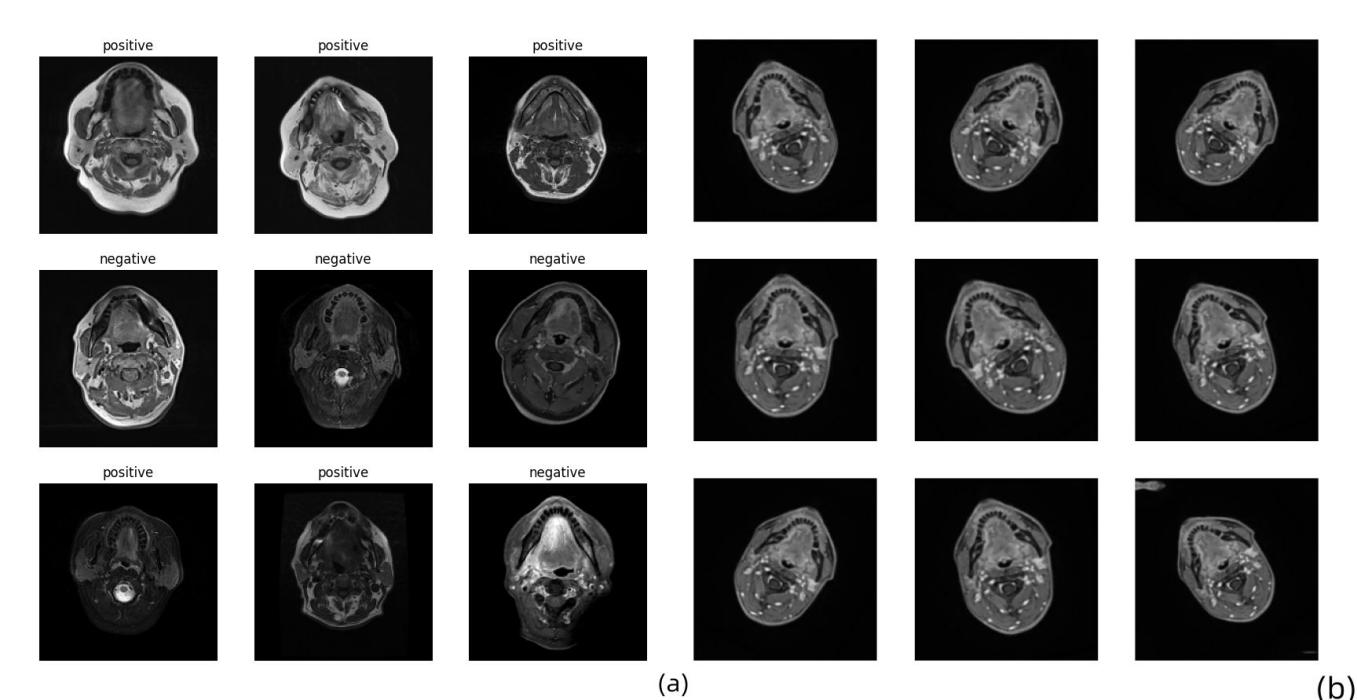


Figure S2. Workflow of the OTSCC lymph node metastasis prediction phase.


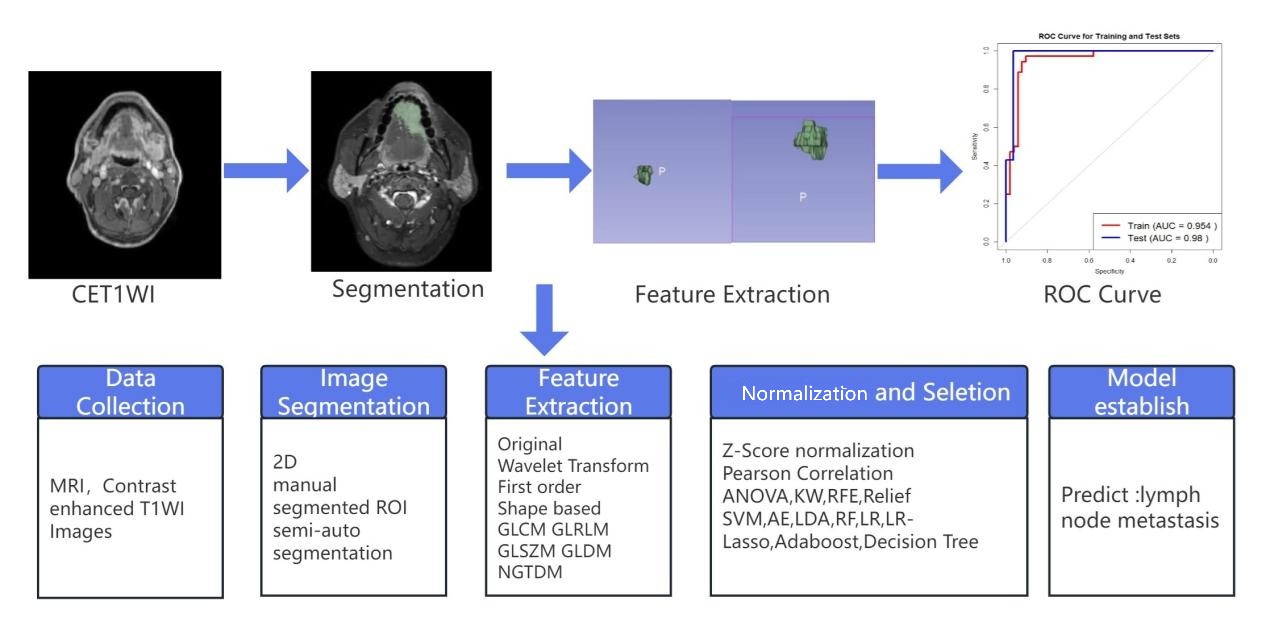


Table S1. Features Selection and Classifier used in our research.

| Models | Features Selection and Classifier |
| --- | --- |
| U-net segmentation |  |
| T1WI | PCC,AE,RFE |
| T2WI | PCC,SVM,RFE |
| CET1 | PCC,AE,KruskalWallis |
| T1WI+T2WI+CET1 | PCC,AE, ANOVA |
| Manual segmentation |  |
| CET1 | PCC,AE,ANOVA |

Pearson Correlation Coefficient (PCC), Analysis of variance (ANOVA), Auto-encoder (AE), Support vector machine (SVM), Recursive feature elimination (RFE).

Figure S3 Auto segmented radiomics with multiple parameters featues rank.


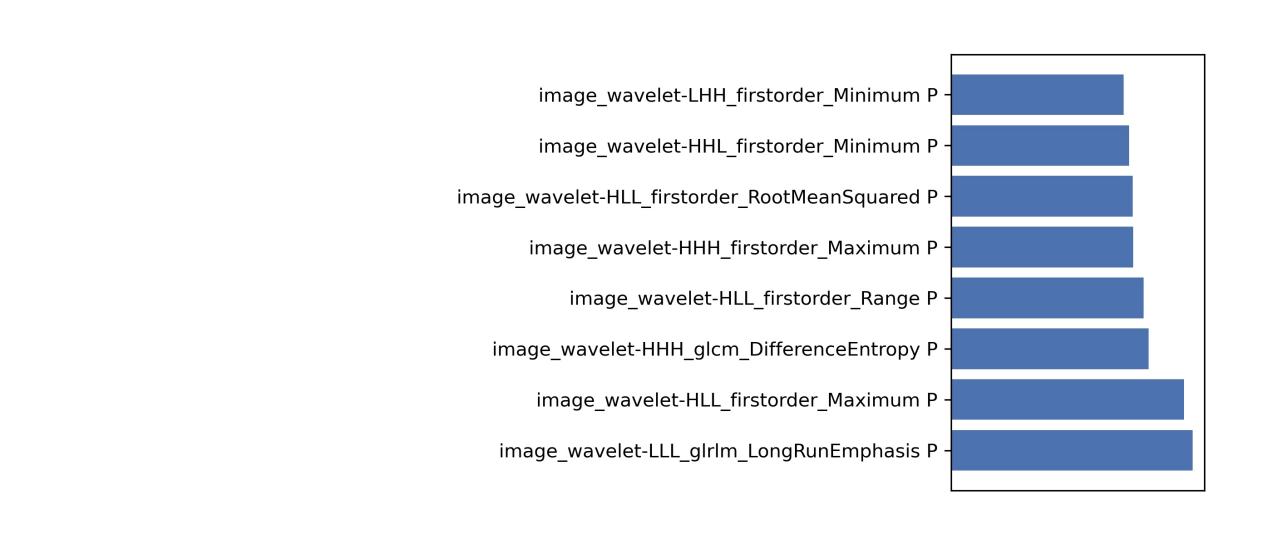


Figure S4 Auto segmented radiomics with CET1 sequence featues rank.


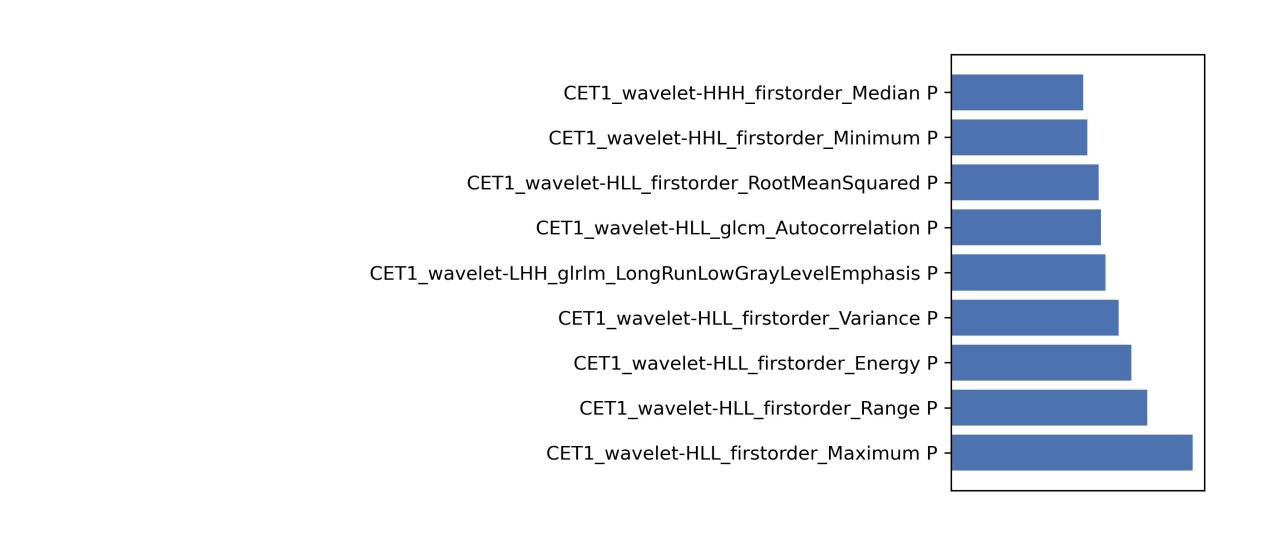


Figure S5 Auto segmented radiomics with T2WI featues rank.


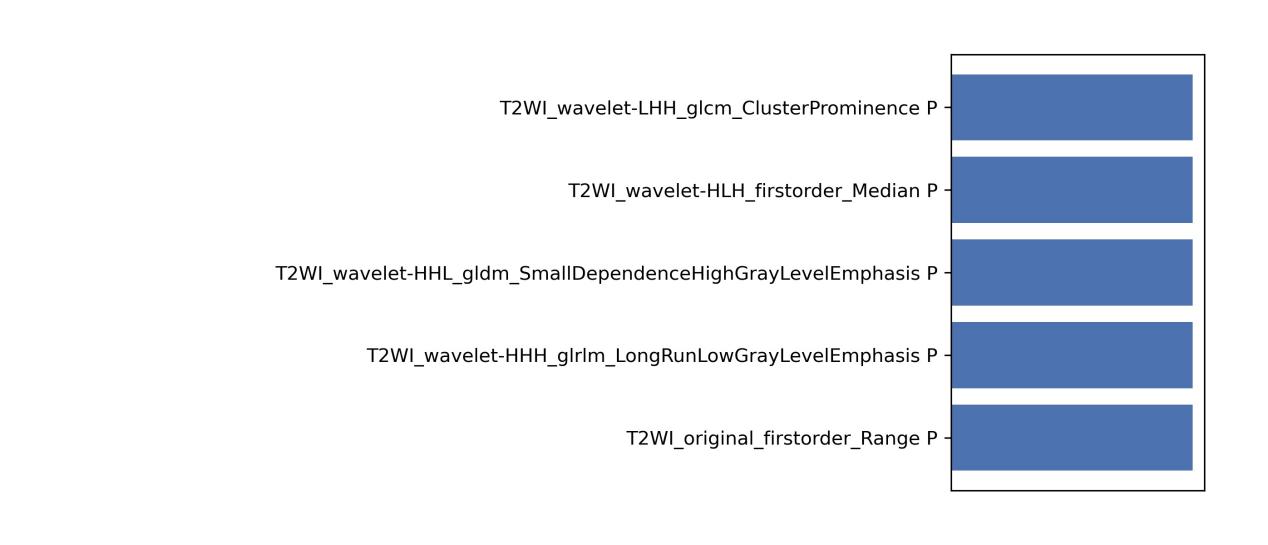


Figure S6 Auto segmented radiomics with T1WI featues rank.


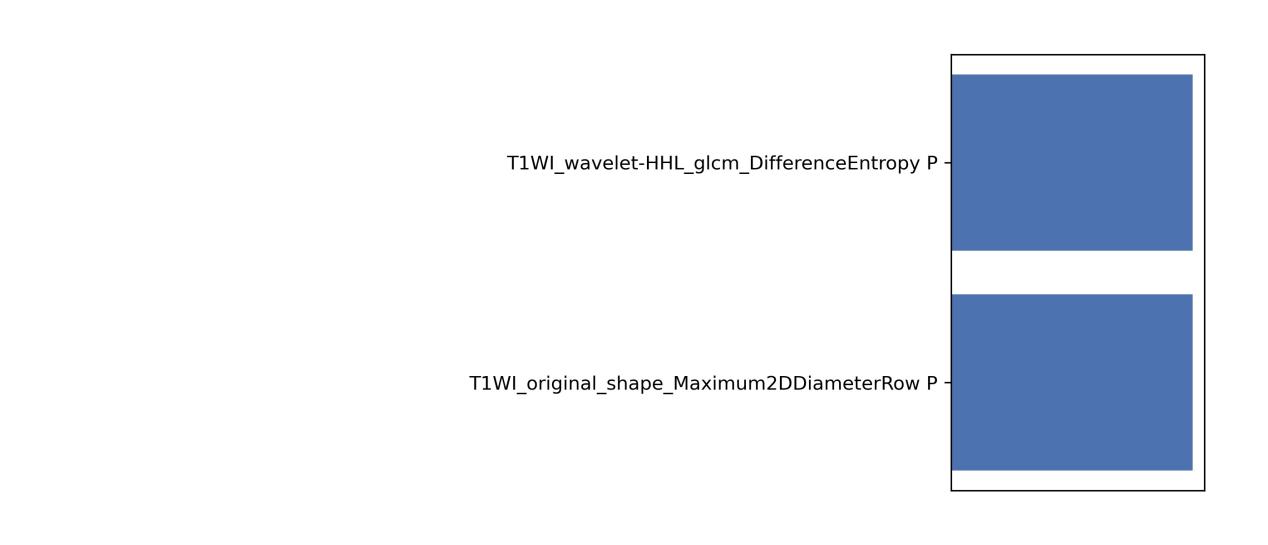


Figure S7 Manual segmented radiomics with CET1 featues rank.


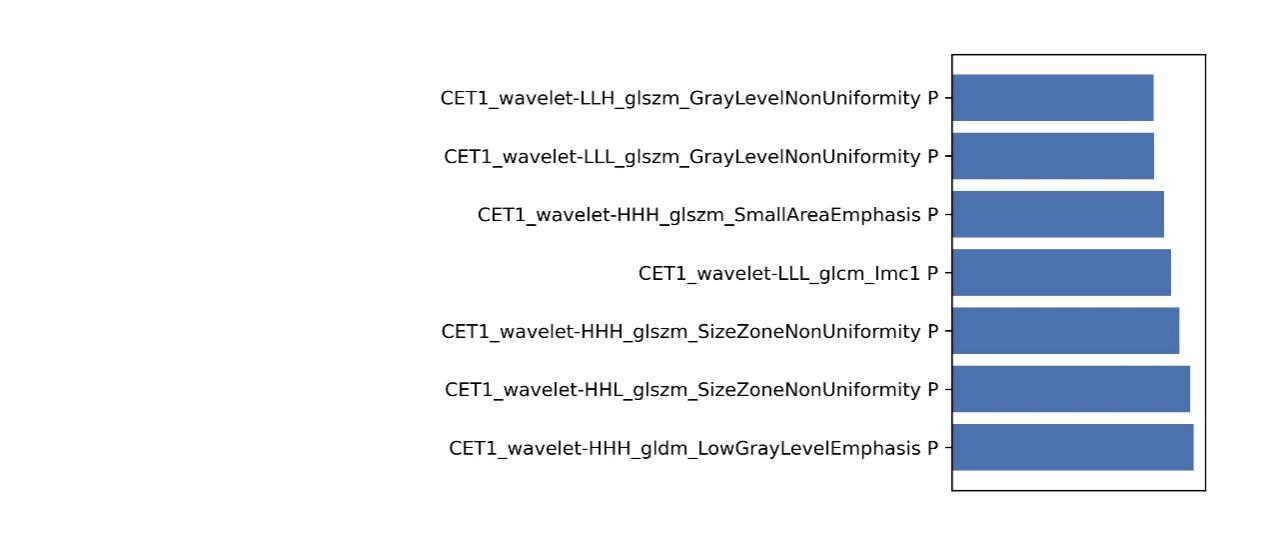


MRI imaging protocol

MRI imaging was performed using various high-field scanners, including PHILIPS Ingenia 3.0T, GE HDxt 3.0T, PHILIPS Achieva Tx 3.0T, GE Signa HDx Series 1.5T, and SIEMENS Prisma 3.0T. Imaging protocols were tailored to each system to optimize diagnostic quality. For the PHILIPS Ingenia 3.0T, T1W sequences used Turbo Spin Echo (TSE) with TR 553 ms and TE 13 ms, while T2W images utilized mDixon sequences (TR 2500 ms, TE 90 ms). CET1 (CET1) was captured with mDixon sequences (TR 610 ms, TE 16 ms). The GE HDxt 3.0T employed T1W imaging with TR 600 ms and TE 9.5 ms, T2 Iterative Decomposition water-fatseparation with Echo Asymmetry and Least-squares estimation (IDEAL) sequences (TR 4180 ms, TE 68 ms), and CET1 with IDEAL (TR 400 ms, TE 10 ms). For the PHILIPS Achieva Tx 3.0T, T1 imaging had TR 600 ms and TE 12 ms, while T2 sequences used STIR with TR 6063 ms, TI 220 ms, and TE 70 ms. The GE Signa HDx Series 1.5T used FSE for T1 (TR 400 ms, TE range 14.3-20.6 ms) and T2 imaging (TR 5000 ms, TE 85 ms) with CET1 using FS IDEAL sequences (TR 420 ms, TE range 15.5-46.5 ms). Lastly, the SIEMENS Prisma 3.0T employed Dixon sequences for T2 (TR 2600 ms, TE 72 ms) and CET1 imaging (TR 6.4 ms, TE 2.64 ms), utilizing mDIXON with dual echo times (TE1 1.35 ms, TE2 2.4 ms) for enhanced contrast. Contrast-enhanced scans were performed following the intravenous injection of gadolinium contrast agents, with an acquisition delay of 220 seconds. Field of view (FOV) was consistently set to 200 × 200 mm.
